# The “*ghost sign*”: focal paleness as a novel marker of an inverted colonic diverticulum

**DOI:** 10.1097/j.pbj.0000000000000221

**Published:** 2023-08-03

**Authors:** Vincent Zimmer

**Affiliations:** a Department of Medicine, Knappschaftsklinikum Saar GmbH, Püttlingen, Germany; b Department of Medicine II, Saarland University Medical Center, Saarland University, Homburg, Germany

## To the Editor:

**Figure 1.**
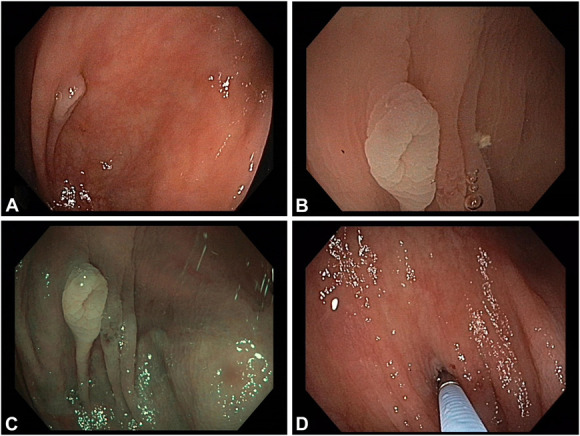
(A) At scope advancement during colonoscopy, a pale elevated lesion was detected behind a fold in the ascending colon. (B) More detailed characterization during withdrawal indicated normal mucosal appearance and a central umbilication on white light endoscopy. (C) Similarly, narrow-band imaging (NBI) did not indicate alterations in mucosal and/or vessel pattern, while reproducing a marked color change compared with the background mucosa. (D) After cautious “*deinversion*,” the lesion was unequivocally confirmed as an inverted diverticulum.

A 59-year-old male patient presented for surveillance colonoscopy on an outpatient basis. During scope advancement, besides a multitude of inconspicuous diverticula in the sigmoid with only occasional typical diverticula in the higher colon, a pale elevated lesion was noted behind a fold in the ascending colon (Fig. 1A). Further characterization on withdrawal indicated a normal mucosal appearance and a central umbilication on white light (Fig. 1B) and narrow-band imaging (Fig. 1C), likewise, however, highlighting the change in color in comparison with the background mucosa. To unequivocally demonstrate the nature of the lesion as an inverted diverticulum, we cautiously “*deinverted*” the diverticulum with the tip of a standard biopsy forceps^1^ (Fig. 1D).

Albeit not per se mutually exclusive, clear-cut differentiation of polys from polypoid inverted colonic diverticula (ICD) as their typical endoscopic presentation is usually straightforward and essential to avoid undue endoscopic resection with inadvertent perforation, entailing potentially serious medical and medico-legal sequelae.^2^ Some endoscopic features, such as surrounding *Aurora* rings, have been reported to this end. Of interest, minor lamina propria edema has been described in mucosal biopsies at the edge of ICD lesions with similar endoscopic presentation.^3^ By contrast, a change in color to a paler, ghost-like appearance, however, most likely attributable to the different wall composition and/or prolonged mechanical stress, has not been explicitly highlighted in the literature before. Notwithstanding, to fully assess the diagnostic potential of what might be designated the “*ghost sign*,” systematic studies are warranted.^3^
